# Yizong Tongluo formula attenuates idiopathic pulmonary fibrosis and inflammatory injury by inhibiting HIF-1α/LSH/SCD1-mediated ferroptosis

**DOI:** 10.3389/fimmu.2026.1760615

**Published:** 2026-02-11

**Authors:** Xin Ma, Xin Wang, Daobin Jiang, Yiminnuer Tuerxun, Siming Tao, Fengsen Li, Yanrong Wei, Liu Yang, Lei Xi, Ling Wang

**Affiliations:** 1 The Fourth Clinical Medical College, Xinjiang Medical University, Urumqi, China; 2 Department of Respiration, Traditional Chinese Medicine Hospital of Xinjiang Uygur Autonomous Region, Urumqi, China

**Keywords:** ferroptosis, HIF-1α/LSH/SCD1 axis, idiopathic pulmonary fibrosis, inflammatory injury, Qi deficiency and blood stasis, Yizong Tongluo formula

## Abstract

**Background:**

Idiopathic pulmonary fibrosis (IPF) is a progressive disease characterized by chronic inflammation, aberrant tissue remodeling, and hypoxia. In traditional Chinese medicine (TCM), it is classified as Qi deficiency–blood stasis syndrome. The mechanism of Yizong Tongluo Formula (YZTLF), a Traditional Chinese Medicine (TCM) herbal formulation and effective pharmaceutical agent for the clinical treatment of IPF, remains unclear.

**Methods:**

This study investigated the immunomodulatory and anti-fibrotic mechanisms of YZTLF for this IPF subtype by focusing on the HIF-1α/LSH/SCD1 signaling pathway and ferroptosis-induced inflammatory injury. We began by analyzing clinical samples from IPF patients with Qi deficiency and blood stasis was conducted to assess the signalling axis and ferroptosis markers. Molecular and histological analyzes were performed using TGF-β-treated MRC-5 fibroblast cells and a bleomycin-induced rat model of pulmonary fibrosis.

**Results:**

Clinical analysis revealed a dysregulation of the HIF-1α/LSH/SCD1 axis and altered levels of ferroptosis markers in patients. *In vitro*, YZTLF significantly downregulated HIF-1α and LSH expression while upregulating SCD1 (*p < 0.01*). Importantly, the treatment markedly suppressed ferroptosis, as evidenced by reduced intracellular Fe^2+^ and ACSL4 levels alongside increased Glutathione Peroxidase 4 (GPX4) and GSH expression (*p < 0.01*). It also inhibited TGF-β-induced fibroblast activation, significantly decreasing α-SMA and Collagen I protein levels (*p < 0.01*). *In vivo*, the YZTLF treatment attenuated bleomycin-induced lung injury, reduced inflammatory cell infiltration, preserved alveolar architecture, and reduced collagen deposition, alongside normalizing of HIF-1α/LSH/SCD1 signaling and restoration of antioxidant levels.

**Conclusion:**

These findings indicate that YZTLF mitigates IPF progression in the context of Qi deficiency and blood stasis by suppressing ferroptosis-driven inflammation and remodeling the hypoxic microenvironment via the HIF-1α/LSH/SCD1 pathway. This provides a mechanism-based rationale for its use in this TCM-defined IPF subtype.

## Introduction

Idiopathic pulmonary fibrosis (IPF) is a chronic lung disease of unknown etiology, characterized by progressive and irreversible scarring of the lungs. Consequently, this condition results in a continual decline in respiratory function and a poor prognosis (1). Globally, IPF affects approximately 3 million people, with incidence and prevalence rates on a consistent upward trend, ranging from 0.09 to 1.30 per 10, 000 individuals and continuing to rise (2). South Korea, the United States, and Canada report the highest incidence rates. Currently, pirfenidone (an anti-inflammatory and anti-fibrotic agent) and nintedanib (a multi-target tyrosine kinase inhibitor) are the only approved treatments to slow pulmonary function decline (50% reduction in annual FVC decline). However, neither agent reverses fibrosis, and both can cause gastrointestinal adverse effects such as nausea and diarrhea (3, 4). This highlights the potential clinical value of Traditional Chinese Medicine (TCM), which has lower toxicity profiles, for managing IPF.

Traditional Chinese Medicine (TCM) classifies IPF into distinct syndromes, including primary syndromes like Yin deficiency with lung dryness, lung Qi deficiency, and lung-kidney Qi deficiency. Concurrent syndromes such as phlegm-dampness and blood stasis, as well as other common patterns like Qi-Yin deficiency, spleen-kidney Yang deficiency, and phlegm-heat obstructing the lung, are also recognized (5, 6). Most IPF cases present with deficiency syndromes alongside concurrent excess patterns, notably Qi deficiency-blood stasis syndrome. In modern medicine, “Qi deficiency” is associated with a weakened immune system and reduced cellular energy, while “blood stasis” indicates poor blood circulation, increased blood clotting, and a lack of oxygen in the tissues. Clinical features include fatigue, shortness of breath, spontaneous sweating (a key sign of Qi deficiency), a sallow complexion, and a cyanotic tongue with ecchymosis (indicative of blood stasis) (7). Therapeutic modalities encompass herbal formulations (Buyang Huanwu Decoction, Xuefu Zhuyu Decoction, and Yizong Tongluo Formula) and acupuncture (8). Emerging evidence suggests that YZTLF exerts unique therapeutic benefits in Qi deficiency-blood stasis IPF by reducing the risk of acute exacerbations, improving core TCM symptoms, and stabilizing the decline in pulmonary function (9).

YZTLF is composed of several Chinese medicinal herbs, including *Astragalus membranaceus*, *Codonopsis pilosula*, *Rhodiola rosea*, and *Cimicifuga foetida*. *Astragalus membranaceus* enhances hemodynamics, alleviates vascular inflammation, and alleviates oxidative stress, thereby protecting vascular integrity (10, 11). *Codonopsis pilosula* polysaccharide has demonstrated efficacy in ameliorating spleen deficiency in murine models through gut microbiota modulation and energy metabolism regulation (12). *Rhodiola rosea* exerts anti-inflammatory, antioxidant, and immunomodulatory effects, showing therapeutic potential for chronic respiratory diseases (13, 14). We hypothesize that these bioactive constituents likely act synergistically on multiple targets to treat Qi deficiency-blood stasis IPF.

These bioactive components may treat Qi deficiency and blood stasis-associated IPF by synergistically acting on multiple targets. Because “blood stasis” results in poor blood flow and hypoxia, we hypothesized that YZTLF’s molecular mechanism involves regulating hypoxia-related pathways. The cellular concentration of HIF-1α, an 826-amino acid protein and the oxygen-sensing subunit of the HIF-1 complex, is tightly regulated by oxygen availability. Under normal oxygen conditions (normoxia), prolyl hydroxylase domain (PHD) enzymes hydroxylate HIF-1α, marking it for continuous proteasomal degradation via VHL-mediated ubiquitination. Conversely, under low-oxygen (hypoxic) conditions, the activity is suppressed, allowing HIF-1α to accumulate, translocate to the nucleus, and initiate gene transcription (15). This process is relevant in specific forms of IPF associated with Qi deficiency and blood stasis, where impaired circulation diminishes oxygen supply, potentially leading to HIF-1α accumulation (16). Moreover, research suggests that HIF-1α can directly activate lymphocyte-specific helicase (LSH) through a mechanism that depends on EGLN1 and c-Myc (17). LSH epigenetically represses stearoyl-CoA desaturase-1 (SCD1), which inhibits ferroptosis via reduced lipid peroxidation (18). Interestingly, SCD1-generated monounsaturated fatty acids (MUFAs) can stabilize HIF-1α through feedback regulation (19). The term “ferroptosis” was coined to describe this specific form of iron-dependent cell death, deriving from the Latin ferrum (iron) and the Greek ptosis (meaning ‘a fall’), with the suffix chosen to align with ‘apoptosis’ and designate a distinct pathway of regulated cellular demise. As ferroptosis critically contributes to alveolar epithelial injury in IPF, elucidating the interplay between HIF-1α, LSH, and SCD1 is essential for exploring YZTLF’s mechanism of action (20–22).

By integrating traditional Chinese medicine (TCM) principles with modern biomedical approaches, this study investigates the therapeutic mechanism of YZTLF for IPF with Qi deficiency and blood stasis. Initial analysis of clinical samples from patients with this IPF subtype revealed altered expression of HIF-1α, LSH, and SCD1, alongside ferroptosis-related proteins Acyl-CoA synthetase long-chain family member 4 (ACSL4) and Fe^2+^ changes, prompting further investigation. Subsequent cellular and animal experiments validated the existence and regulatory hierarchy of the HIF-1α/LSH/SCD1 pathway. YZTLF suppresses ferroptosis via this pathway, demonstrating its therapeutic efficacy in mitigating IPF progression.

## Materials and methods

### Clinical sample collection and processing

The study protocol received ethical approval from the Ethics Committee of Traditional Chinese Medicine Hospital of Xinjiang Uygur Autonomous Region (Approval No: 2025XE-GS231) and was conducted in accordance with the principles outlined in the Declaration of Helsinki. All participants provided written informed consent before their enrollment in the study, after which peripheral blood samples were drawn. Participants were then categorized into three groups: healthy individuals (Control group, n=6), patients diagnosed with IPF presenting with Qi deficiency and blood stasis syndrome (Patient group, n=6), and IPF patients with the same TCM syndrome who had completed a course of treatment with YZTLF (YZTLF group, n=6). YZTLF composition was shown in **Table 1**. Diagnosis of IPF followed the official ATS/ERS/JRS/ALAT clinical practice guidelines. TCM syndrome differentiation (Qi deficiency and blood stasis) was confirmed by two senior TCM clinicians in accordance with the Guiding Principles for Clinical Research of New Chinese Medicines, based on characteristic symptoms including fatigue, shortness of breath, spontaneous sweating, sallow complexion, and a cyanotic tongue with ecchymosis. Patients were enrolled based on the following inclusion criteria: (1) age ≥ 40 years; (2) a confirmed or highly suspected diagnosis of IPF according to ATS/ERS/JRS/ALAT criteria within the preceding 5 years, validated by high-resolution computed tomography (HRCT) or lung biopsy within 12 months prior to enrollment; (3) pulmonary function characterized by a forced vital capacity (FVC) ≥ 50% of the predicted value and a diffusing capacity for carbon monoxide (DLCO) between 30% and 79% of the predicted value; and (4) concordance with the TCM diagnostic criteria for Qi deficiency and blood stasis syndrome. Patients were excluded if they presented with severe hepatic, renal, or cardiac dysfunction (NYHA class III–IV), malignancies, or coagulation disorders. Additionally, individuals currently using immunosuppressants or glucocorticoids, or those participating in other clinical trials, were excluded from the study.

**Table 1:** YZTLF composition.

English name	Chinese name	Latin name	Part used	Dosage
Astragali Radix	Huangqi	*Astragalus membranaceus* (Fisch.) Bge. var. mongholicus (Bge.) Hsiao	Root	30g
Codonopsis Radix	Dangshen	*Codonopsis pilosula* (Franch.) Nannf.	Root	15g
Rhodiolae Crenulatae Radix et Rhizoma	Hongjingtian	*Rhodiola crenulata* (Hook. f. et Thoms.) H. Ohba	Root and Rhizome	15g
Cimicifugae Rhizoma	Shengma	*Cimicifuga foetida* L.	Rhizome	6g
Bupleuri Radix	Chaihu	*Bupleurum chinense* DC.	Root	6g
Platycodonis Radix	Jiegeng	*Platycodon grandiflorus* (Jacq.) A. DC.	Root	6g
Luffae Fructus Retinervus	Sigualuo	*Luffa cylindrica* (L.) Roem.	Vascular Bundle	6g
Curcumae Rhizoma	Ezhu	*Curcuma phaeocaulis* Val.	Rhizome (Vinegar-processed)	6g
Angelicae Sinensis Radix	Danggui	*Angelica sinensis* (Oliv.) Diels	Root	15g
Anemarrhenae Rhizoma	Zhimu	*Anemarrhena asphodeloides* Bunge	Rhizome	10g

Samples of whole blood were drawn into vacuum EDTA tubes and subsequently processed within a two-hour window. For serum isolation, the samples were incubated at ambient temperature for 30 minutes to permit coagulation, after which they were centrifuged for 15 minutes at 3, 000×g and 4 °C. The resulting serum supernatant was then carefully pipetted into sterile cryovials and promptly frozen at -80 °C to await subsequent analysis.

### Preparation of Yizong Tongluo formula

The Chinese herbal medicines were placed in a clay pot and soaked in tap water for 20 minutes. The mixture was brought to a boil and simmered for 30 minutes, after which the liquid was filtered. For the second decoction, water was added to the residue, boiled, and simmered for 25 minutes before filtering. The two filtrates were combined to obtain the final decoction for clinical trials. For animal experiments and *in vitro*, the decoction was concentrated and adjusted to a standard physiological pH (7.2–7.4), and filtered through a 0.22 μm sterile membrane to obtain the stock solution. Subsequent CCK-8 assays confirmed that the working concentration maintained cellular viability, ruling out non-specific cytotoxicity caused by physicochemical alterations.

### Cell culture and treatment

Human lung fibroblast MRC-5 cells were grown in Dulbecco’s Modified Eagle Medium (DMEM) that contained 10% fetal bovine serum (FBS) and 1% penicillin/streptomycin. These cultures were kept in or maintained at 37 °C in a 5% CO_2_ atmosphere. When the cell density reached 80-90%, they were passaged with a 0.25% trypsin-EDTA solution.

To establish an *in vitro* model of IPF, fibrotic activation was induced by treating the MRC-5 cells with 5 ng/mL of recombinant human TGF-β1 (R&D Systems) for a duration of 48 hours (23) experimental groups were designated as follows: Normal control (NC, untreated MRC-5 cells), Model (TGF-β1-induced cells), si-NC (siRNA negative control), siHIF-1α (Model + 50 nM HIF-1α-targeting siRNA), YZTLF (Model + 200 mg/L YZTLF), and Combination (Model + YZTLF + si-HIF-1α). YZTLF decoction, sourced from the Traditional Chinese Medicine Hospital of Xinjiang Uygur Autonomous Region, was first centrifuged for ten minutes at 12, 000 ×g. For sterilization, the resulting liquid was subsequently passed through a 0.22-μm filter. This sterile supernatant was then brought to its final working concentration of 1, 000 mg/L (total extract) by dilution with DMEM before being administered to the cells for 24 hours. The transfection of siRNA into the cells was accomplished with the Lipofectamine 3000 reagent (Thermo Fisher Scientific).

For an investigation into LSH-mediated regulation of SCD1 by HIF-1α, several experimental cohorts were prepared. These included a Model cohort, si-NC (Model + si-NC), si-HIF-1α (Model + HIF-1α siRNA), oe-NC (Model + empty vector), oe-LSH (Model + LSH overexpression plasmid), and a combination of si-HIF-1α + oe-LSH (Model + HIF-1α siRNA + LSH plasmid). Each well received 2 µg of plasmid DNA for transfection. All experimental conditions were conducted in triplicate, and for subsequent analysis, cellular materials were collected 48 hours after the treatments.

### Animals

All experimental procedures involving animals were conducted in compliance with the guidelines of Regulations on the Administration of Laboratory Animals (State Council of China), and received approval from Animal Ethics Committee of Jiangxi Hengqing Testing Technology Co., Ltd. (Approval No: JXHQ2025005). Male Sprague-Dawley rats, weighing between 200–250 g, were used for this study. The animals were housed in a controlled environment under a 12-hour light/dark cycle with a stable room temperature of 22 ± 1 °C and a relative humidity of 50 ± 10%. Standard rodent chow and water were available ad libitum for the duration of the study.

To induce a model of pulmonary fibrosis, isoflurane was used to anesthetize the rats, which were then placed in a supine position. The neck area was shaved and sterilized with ethanol before a midline cervical incision was performed to reveal the trachea. A 1-mL syringe was utilized to slowly administer a 5 mg/kg bleomycin solution directly into the trachea, with the needle inserted between the cartilage rings at a near-parallel angle. Post-injection, the wound was sutured, and rats were held upright for 1–3 minutes while gently rotating and massaging the thorax to ensure homogeneous distribution of bleomycin. Animals were monitored postoperatively for recovery and maintained for 28 days before tissue and blood collection.

The rats were randomly assigned to the following experimental groups (*n=6* per group), including Control (healthy rats, no treatment), Model (bleomycin-induced IPF), YZTLF-L (Model + low-dose YZTLF, 5 g/kg/day), YZTLF-M (Model + medium-dose YZTLF, 10 g/kg/day), YZTLF-H (Model + high-dose YZTLF, 20 g/kg/day), and Model + nintedanib (0.05 g/kg/day). YZTLF, prepared as a decoction using traditional methods, was filtered (0.22-μm pore) and diluted in distilled water for daily oral gavage (5 mL/kg). Nintedanib (MedChemExpress) was suspended in 0.5% carboxymethyl cellulose and administered via the same route. Treatments commenced 24 hours post-bleomycin instillation and continued for 28 days. Post-experiment, lung tissues and serum were collected for histopathological and biochemical analyzes.

The dosage was calculated based on the body surface area normalization method, using the standard conversion factor of 6.3 between humans and rats (Rat dosage = Human dosage × 6.3), to ensure clinical relevance.

### Chromatin immunoprecipitation

ChIP was performed to assess the binding of HIF-1α and LSH to specific DNA regions. Initially, MRC-5 cells were cross-linked through a 10-minute incubation with 1% formaldehyde at ambient temperature; this process was quenched by adding 0.125 M glycine. After a PBS wash, the cells underwent lysis using a designated ChIP lysis buffer. The resulting chromatin was then fragmented into 200–1000 base pair segments via sonication. The immunoprecipitation step was performed by incubating the fragmented chromatin overnight at 4 °C. One of the following specific primary antibodies was used for each reaction: Anti-HIF-1α (Proteintech, 20960-1-AP), Anti-LSH (Santa Cruz Biotechnology, c-365815X), or a Normal rabbit IgG (CST, 2729) negative control. The immune complexes were subsequently isolated from the solution using Protein A/G magnetic beads. Following washes and cross-link reversal, DNA was purified with a commercial kit (QIAGEN, 28104). Analysis of the purified DNA was conducted via electrophoresis on a 1.5% agarose gel, with the bands being documented using a gel imaging system (Bio-Rad ChemiDoc MP). Binding was confirmed by the enrichment of specific DNA bands in the experimental samples relative to the IgG control.

### Dual-Luciferase reporter assay

To assess the regulatory effect of LSH on the activity of the SCD1 promoter, specific reporter constructs were developed. Fragments of the SCD1 promoter (–1500 to +100 bp) for both wild-type (WT) and mutant (MT) versions were inserted into the pGL3-Basic vector (Promega). The mutant construct was created using site-directed mutagenesis, which was designed to interrupt the putative binding sites for HIF-1α/LSH.

MRC-5 cells were co-transfected with a combination of plasmids: 500 ng of either the WT- or MT-pGL3 reporter, 50 ng of the pRL-TK control plasmid, and 1 µg of either an LSH overexpression plasmid or an empty vector. The transfection procedure was carried out with Lipofectamine 3000 (Thermo Fisher). After 48 hours, the resulting reporter activity was measured using the Dual-Luciferase Reporter Assay System (Promega). To control for variations in transfection efficiency across samples, the signal from firefly luciferase was adjusted by normalizing it to the corresponding Renilla luciferase signal.

### Cell counting kit-8 assay

The viability of MRC-5 cells was assessed using a CCK-8 assay. Initially, cells were plated in 96-well plates at a concentration of 5 × 10^4^ per well and grown to their logarithmic phase. YZTLF drug was sterilized by filtration through a 0.22-µm membrane to remove any particulates. A dilution series of the drug was then created, yielding concentrations of 0, 10, 50, 100, 200, and 400 mg/L. Cultures were treated with this range of concentrations for 24 hours. After the treatment period, 10 µL of CCK-8 reagent was introduced to each well, and the plates were subjected to a final two-hour incubation at 37 °C. Viability was subsequently quantified by reading the absorbance at 450 nm on a microplate reader.

### The detection of iron ion, GSH, and MDA level

The Iron Assay Kit (Colorimetric, Abcam, ab83366) was used to measure Fe^2+^ levels. For sample preparation, particulate matter was first pelleted from serum and cell supernatants via centrifugation (3000 × g for 15 minutes at 4 °C). To reduce matrix effects, samples were subsequently diluted 1:10 with the kit’s buffer. The final iron concentration was calculated based on the absorbance reading at 593 nm, measured on a BioTek Synergy H1 microplate reader, and compared against a standard curve of iron standards (0–100 µM).

The concentration of GSH was measured with a Glutathione Assay Kit (Sigma-Aldrich, MAK259) based on a kinetic enzymatic recycling method. To begin, samples were deproteinized using a 1:1 volume of 5% sulfosalicylic acid and then centrifuged (10, 000 × g for 10 minutes) to clarify. The resulting supernatant was combined with 5, 5′-dithiobis (2-nitrobenzoic acid) (DTNB) and glutathione reductase. The reaction progress was tracked by monitoring the change in absorbance at 412 nm. Final GSH values were determined by comparison to a standard curve (0–20 µM) and then adjusted based on the total protein content of each sample, which was measured independently with a BCA assay (Thermo Fisher, 23225).

To assess lipid peroxidation, malondialdehyde (MDA) levels were quantified with a TBARS Assay Kit (Cayman Chemical, 10009055). In the procedure, samples were combined with thiobarbituric acid (TBA) and incubated at 95 °C for 60 minutes to facilitate the formation of the MDA-TBA adduct. After the mixture cooled, it was centrifuged for 10 minutes at 1600 × g. The absorbance of the resulting supernatant was then measured at 532 nm. Quantification was achieved by comparing the results to a standard curve generated from 1, 1, 3, 3-tetramethoxypropane (0–50 µM).

### Reactive oxygen species detection

A fluorescence-based flow cytometry assay was employed to measure intracellular ROS. For this purpose, cells were labeled with 10 µM of the ROS-sensitive fluorescent probe DCFH-DA (Beyotime, S0033). This labeling step was carried out in the dark for 30 minutes at 37 °C using serum-free medium. After the incubation, cells were rinsed two times with phosphate-buffered saline (PBS; HyClone, SH30256.01) and resuspended in warm PBS. Analysis was promptly performed on a BD FACSCanto II flow cytometer (BD Biosciences). The fluorescent signal was detected with an excitation of 488 nm and emission of 525 nm. FlowJo software (v10.8.1) was used for data processing, with gating based on an unstained negative control.

### Apoptosis detection

The extent of cellular apoptosis was determined with an Annexin V-FITC/PI Apoptosis Detection Kit (BD Biosciences, 556547). To prepare samples, 1 × 10^6^ cells were collected via mild trypsinization (Thermo Fisher, 25200072), rinsed twice in cold PBS, and resuspended in 100 µL of binding buffer. Cells were then co-stained with 5 µL of Annexin V-FITC and 5 µL of propidium iodide (PI). This labeling was allowed to incubate for 15 minutes at ambient temperature while shielded from light. Before cytometric analysis, each sample was diluted with an additional 400 µL of binding buffer. Data were collected within an hour of staining using the FL1 (530/30 nm) and FL3 (>670 nm) detection channels.

### Western blot

First, cells were lysed on ice using RIPA buffer (Beyotime, ST506) supplemented with 1 mM PMSF to inhibit protease activity. The cell lysates were then clarified by centrifugation at 12, 000 × g for 15 minutes at 4 °C. The total protein content of the resulting clarified supernatant was then precisely determined using a BCA Protein Assay Kit (Thermo Fisher Scientific, 23225). For each sample, 20–40 µg of protein was separated on 8–12% SDS-PAGE gels and then transferred onto PVDF membranes (Millipore, IPFL00010). The membranes were then treated with a blocking solution (5% non-fat milk in TBST) for one hour at ambient temperature to minimize non-specific signals. After blocking, the membranes were probed overnight at 4 °C with one of these primary antibodies: HIF-1α (1:600; Proteintech, 20960-1-AP), LSH (1:800; CST, sc-365815X), SCD1 (1:10000; Proteintech, 10627-1-AP), ACSL4 (1:40000; Proteintech, 22286-1-AP), GPX4 (1:3000; CST, 52455), α-SMA (1:1000; CST, 19245), Collagen I (1:800; CST, 84336), or β-actin (1:50000; Proteintech, 66009-1-Ig). Following a series of washes, the membranes were treated for one hour at ambient temperature with a suitable HRP-conjugated secondary antibody (1:10000; Proteintech, SA00002-2). Protein bands were visualized using an enhanced chemiluminescence (ECL) substrate (Thermo Fisher Scientific, 34580) and imaged using the Tanon-5200 Multi-Automatic Chemiluminescence Imaging Analysis System (Shanghai Tanon Technology Co., Ltd.). Densitometric analysis was performed using ImageJ software (NIH, USA), and the band intensity of target proteins was normalized to that of β-actin.

### Quantitative real-time PCR

For qRT-PCR analysis, TRIzol Reagent (Invitrogen, 15596026) was utilized for the initial extraction of total RNA from cell or tissue samples. A NanoDrop spectrophotometer (Thermo Fisher) was then employed to assess the purity and concentration of the isolated RNA. Following this, first-strand complementary DNA (cDNA) was synthesized from 1 µg of the total RNA template using the PrimeScript RT Reagent Kit (Takara, RR037A). Quantitative PCR was conducted in triplicate on a QuantStudio 5 Real-Time PCR System (Applied Biosystems) with SYBR Green Master Mix (Thermo Fisher, A25742). Thermocycling was performed with the following profile: an initial denaturation step at 95 °C for 10 minutes, followed by 40 cycles alternating between 95 °C for 15 seconds and 60 °C for 1 minute. Primers were designed using the Primer-BLAST tool (NCBI) and were experimentally validated for specificity (**Table 2**); this was additionally confirmed via melt curve analysis after every run. The relative mRNA expression levels were calculated using the *2^-ΔΔCt^* method, where *ΔCt = Ct_target_ - C_t_, _GAPDH_ and ΔΔCt = ΔCt_sample_ - ΔCt_control_.* GAPDH was used as the stable internal reference gene for normalization. Full details of the specific primer sequences used in this study are listed in **Table 2**.

**Table 2:** The primer sequences for qRT-PCR.

Primers	Direction	Sequence (5'→3')
HIF-1α	Forward	5’-GAAAGCGCAAGTCCTCAAAG-3’
	Reverse	5'-TGGGTAGGAGATGGAGATGC-3'
LSH	Forward	5’-CAGCAGCTCCTCAACCTCAC-3’
	Reverse	5’-GCTGCTGGTAGAGGTGCTTC-3’
SCD1	Forward	5’-CTGCGTCTACACCGACTTCC-3’
	Reverse	5'-GCCGTAGTCGTAGTCGTTCC-3'
GAPDH	Forward	5’-GTCTCCTCTGACTTCAACAGCG-3’
	Reverse	5’-ACCACCCTGTTGCTGTAGCCAA-3’

### Hematoxylin and eosin staining

For the H&E staining protocol, paraffin-embedded tissue blocks were initially sectioned to a thickness of 4–5 µm. The resulting tissue sections were then prepared for staining by first undergoing deparaffinization with xylene. Following this step, the sections were rehydrated by passing them through a descending ethanol gradient, which concluded with a final rinse in distilled water. The staining protocol began by submerging the slides in Mayer’s hematoxylin for an eight-minute period, followed by a rinse with tap water. A one-minute counterstain with 1% eosin Y was then applied. After staining, the sections were processed through a dehydration series, cleared, and mounted. A light microscope was used to examine the prepared slides to evaluate general histopathological changes. Histological evaluation and scoring were performed by two independent investigators who were blinded to the experimental group allocations.

### Masson staining

To evaluate tissue fibrosis, tissue was first sectioned at 4–5 µm. The resulting sections were then mounted on glass slides, cleared of paraffin, and rehydrated for staining. The procedure consisted of three main steps: a 10-min immersion in Weigert’s iron hematoxylin, followed by a 10-min immersion in Biebrich scarlet-acid fuchsin, and concluding with a 10-minute differentiation step using phosphomolybdic-phosphotungstic acid. Following this, a five-minute counterstain with aniline blue was applied. After a brief wash in 1% acetic acid, the samples were processed through a dehydration series, cleared in xylene, and finally mounted with a coverslip for analysis. This staining technique allows for the histological assessment of fibrosis by coloring collagen blue, muscle/cytoplasm red, and nuclei black.

### Statistical analysis

For statistical analysis, all *in vitro* experiments were performed with at least three independent biological replicates (*n = 3*). For clinical and *in vivo* experiments, the total sample size was six (*n = 6*) per group. Specifically, Western blot analyses were performed using three randomly selected biological replicates (*n = 3*). Data are presented as the mean ± standard deviation (SD). Prior to parametric analysis, data distribution normality was verified using the Shapiro-Wilk test, and homogeneity of variance was assessed using Levene’s test. To determine the statistical significance of differences between experimental groups, a one-way analysis of variance (ANOVA) was used, with Tukey’s *post-hoc* test applied for multiple comparisons. A result was deemed statistically significant if the p-value was less than 0.05. All graphs and charts were created with GraphPad Prism software (Version 5.0, USA).

## Results

### Protein alterations of the HIF-1α/LSH/SCD1 axis were validated in clinical samples

**Figure 1:**
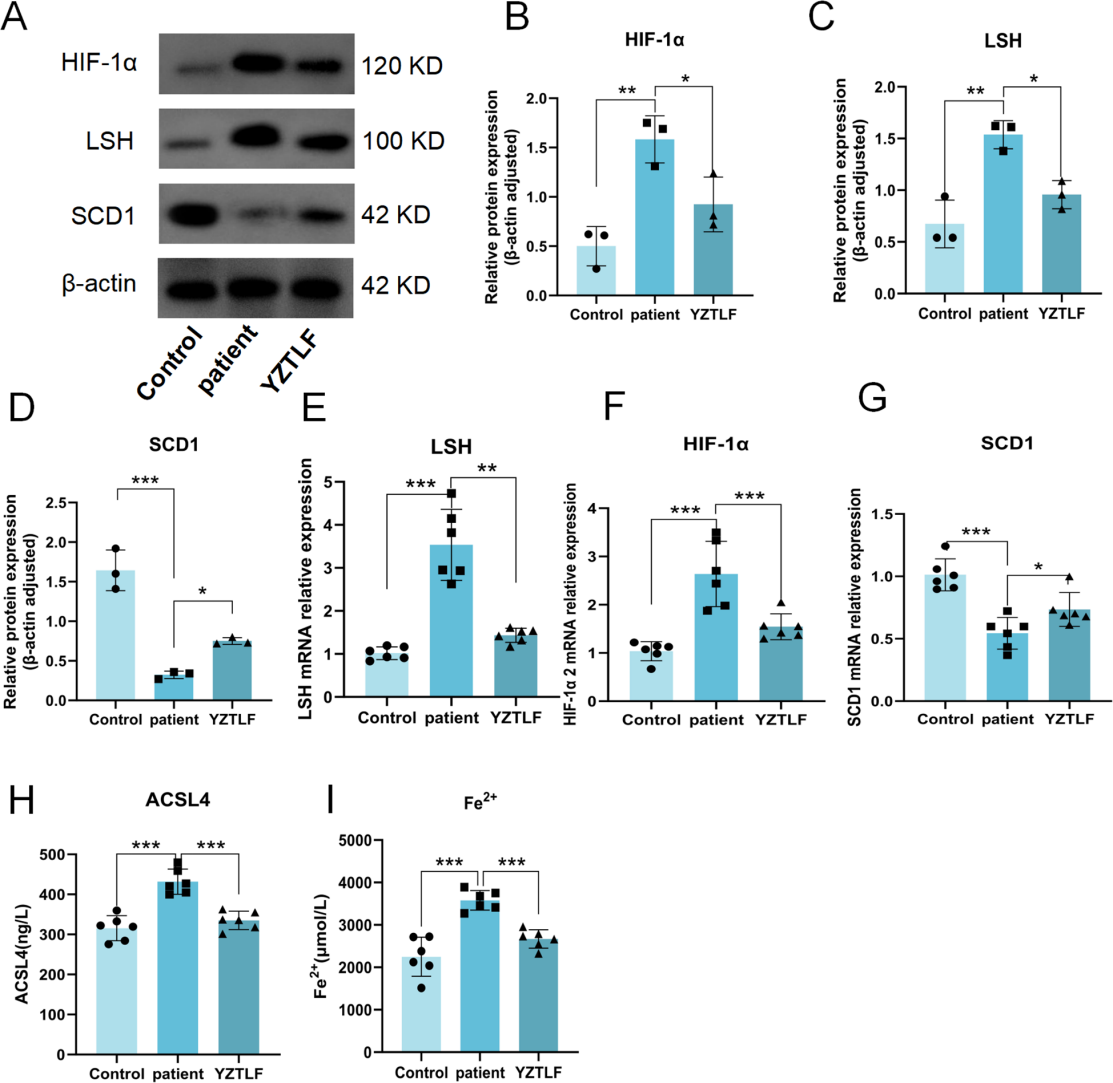
YZTLF modulates the HIF-1α/LSH/SCD1 axis and ferroptosis in clinical samples. **(A)** Representative Western blot images showing the protein levels of HIF-1α, LSH, and SCD1 (*n = 3*). **(B-D)** Quantitative analysis of the protein expression of HIF-1α, LSH, and SCD1. **(E-G)** Relative mRNA expression levels of HIF-1α, LSH, and SCD1, as determined by qRT-PCR. **(H)** Protein expression levels of ACSL4. **(I)** Measurement of intracellular Fe^2+^ levels. Data are presented as mean ± SD. *p < 0.05, **p < 0.01, ***p < 0.001 vs. patient group.

To investigate the regulatory mechanism of the HIF-1α/LSH/SCD1 axis, we first measured the protein and mRNA expression of HIF-1α, LSH, and SCD1 in blood samples from patients and healthy controls. Compared to the healthy control group, the expression levels of both HIF-1α and LSH protein and mRNA were significantly elevated in patients. Following treatment with YZTLF, their expression was markedly reduced. In contrast, the expression of SCD1 protein and mRNA exhibited the opposite trend (**Figures 1A–G**). Furthermore, the ferroptosis-related markers ACSL4 and Fe^2+^ were significantly elevated in patients. YZTLF treatment reduced the levels of both ACSL4 and Fe^2+^, indicating a decrease in the overall level of ferroptosis (**Figures 1H, I**).

### Validation of HIF-1α/LSH/SCD1 interactions

**Figure 2:**
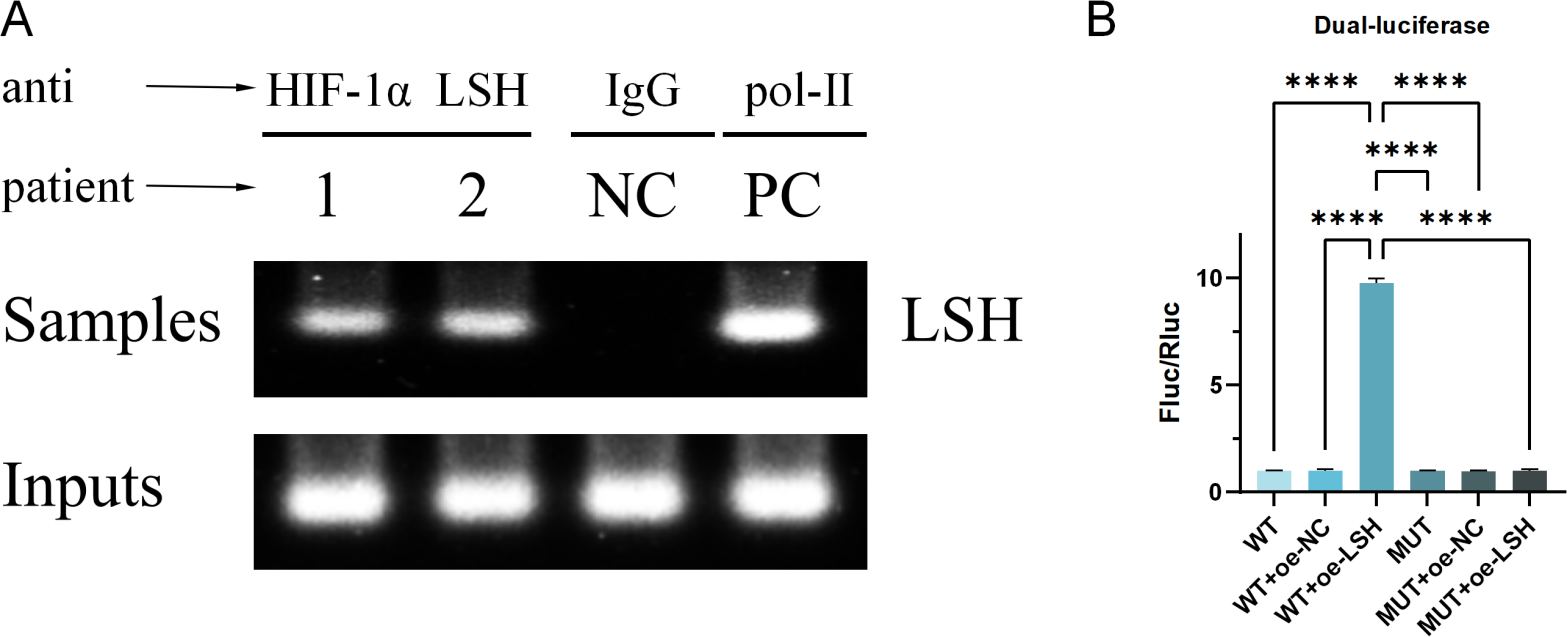
The interactions among HIF-1α, LSH, and SCD1 were validated. **(A)** ChIP assay confirmed the binding interaction between HIF-1α and the DNA region of LSH. **(B)** Dual-luciferase reporter assay demonstrated that LSH regulates SCD1 expression. (*n = 3*) Results are represented as means ± standard deviations. ****p < 0.0001. Comparisons are as indicated by the brackets. Fluc/Rluc indicates firefly luciferase activity normalized to Renilla luciferase.

To elucidate the regulatory relationships among HIF-1α, LSH, and SCD1, ChIP assays were performed to assess HIF-1α-mediated DNA methylation at the LSH locus, followed by dual-luciferase reporter assays to evaluate LSH-dependent SCD1 regulation. ChIP results revealed enrichment of specific DNA fragments encompassing the target region (LSH) in samples immunoprecipitated with anti-HIF-1α and anti-LSH antibodies, while negative (NC) and positive (PC) controls displayed expected patterns, confirming direct binding of HIF-1α to the LSH promoter region (**Figure 2A**). Dual-luciferase assays demonstrated significant Fluc/Rluc ratio elevation upon LSH overexpression in wild-type constructs, with no effect observed in mutant variants, indicating LSH-specific regulation of SCD1 transcription (**Figure 2B**). These findings establish the functional interplay among HIF-1α, LSH, and SCD1, with detailed mechanistic validation provided in subsequent experiments.

### Regulation of Yizong Tongluo formula for ferroptosis via HIF-1α/LSH/SCD1 pathway

**Figure 3:**
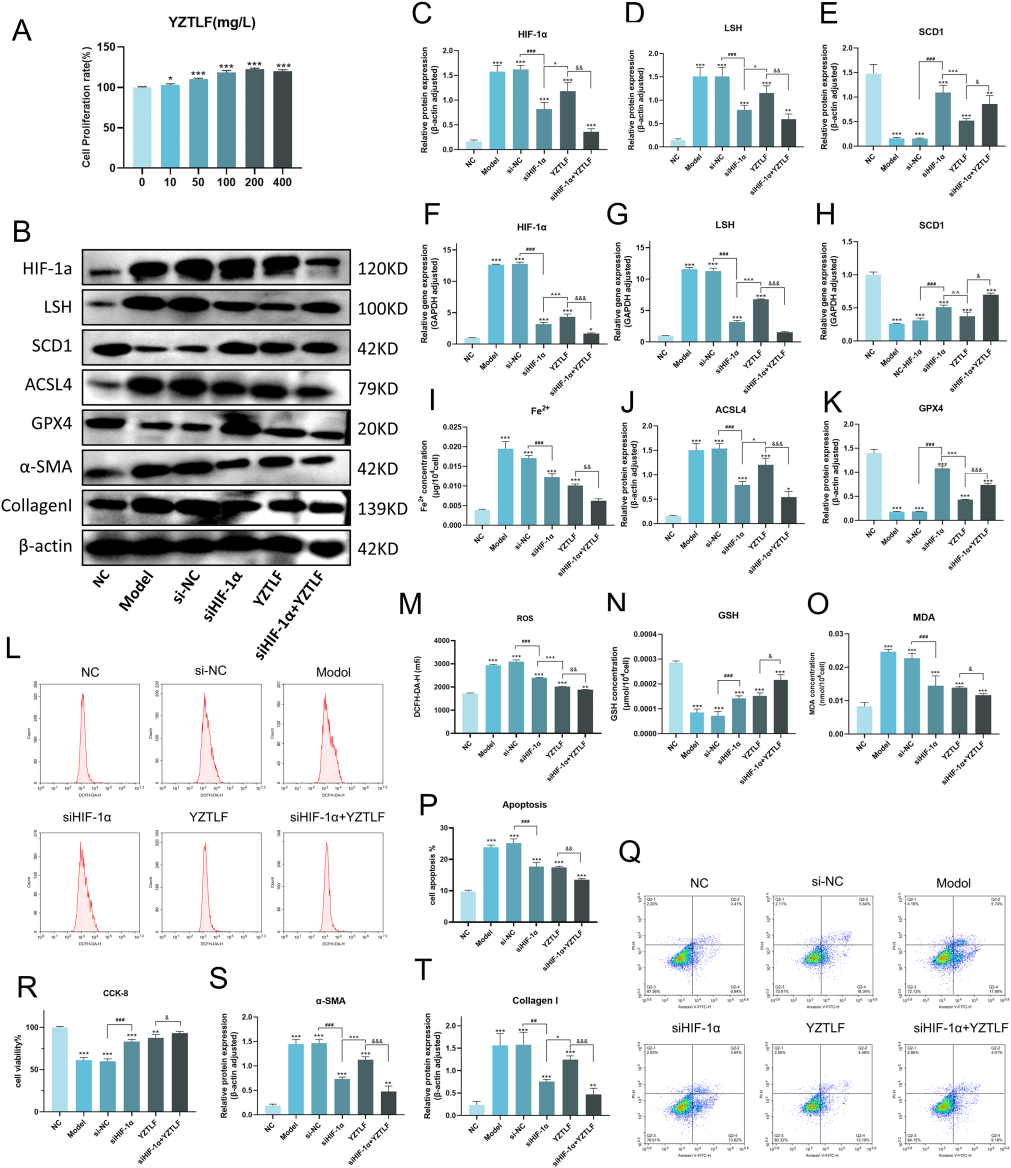
YZTLF modulates the expression of HIF-1α/LSH/SCD1 pathway and ferroptosis-related proteins in cells. NC, normal control. **(A)** Cell viability of MRC-5 cells treated with 0–400 mg/L YZTLF. **(B)** Western blot analysis of a-SMA, Collagen I, HIF-1α, LSH, SCD1, ACSL4, and GPX4 proteins. **(C–E)** Quantification of HIF-1α, LSH, and SCD1 protein levels. **(F–H)** Quantification of HIF-1α, LSH, and SCD1 mRNA expression. **(I)** Ferrous ion (Fe^2+^) levels. **(J, K)** Quantification of ACSL4 and GPX4 protein levels. **(L, M)** ROS levels detected by flow cytometry. **(N, O)** Levels of oxidative stress markers GSH and MDA. **(P, Q)** Cell apoptosis levels detected by flow cytometry. **(R)** Cell proliferation in different treatment groups assessed by CCK-8 assay. **(S, T)** Protein levels of fibrosis markers a-SMA and Collagen **(I)** Results are represented as mean ± SD (*n = 3*). Statistical significance is indicated as follows: *p < 0.05, **p < 0.01, ***p < 0.001 vs. NC group; ^#^*p* < 0.05, ^##^*p* < 0.01, ^###^*p* < 0.001, si-NC vs. siHIF-1α group; ^*p* < 0.05, ^^*p* < 0.01, ^^^*p* < 0.001, YZTLF vs. siHIF-1α group; ^&^*p* < 0.05, ^&&^*p* < 0.01, ^&&&^*p* < 0.001, siHIF-1α+YZTLF vs. YZTLF group.

To investigate whether YZTLF suppresses ferroptosis via the regulation of the HIF-1α-mediated LSH/SCD1 axis, we initially determined the optimal dosage using CCK-8 assay. We identified 200 mg/L as the concentration yielding maximal efficacy without cytotoxicity (**Figure 3A**). Subsequently, TGF-β-induced IPF model was established in MRC-5 cells, accompanied by HIF-1α silence. The data revealed that TGF-β induction significantly upregulated the protein and mRNA levels of HIF-1α and LSH, while concurrently downregulating SCD1 expression. Notably, both HIF-1α silencing and YZTLF treatment significantly reversed these alterations, and the combined intervention demonstrated a synergistic effect (**Figures 3B–H**). To further elucidate the impact of HIF-1α and YZTLF on ferroptosis, we assessed intracellular ferrous iron levels alongside the expression of ferroptosis-related proteins ACSL4 and GPX4. Compared to the model group, both HIF-1α knockdown and YZTLF treatment attenuated ferrous iron accumulation and reduced ACSL4 levels, with the combined treatment yielding better efficacy. Conversely, GPX4, an antioxidant enzyme against ferroptosis, exhibited an inverse trend (**Figures 3I–K**).

Given that oxidative stress is a critical driver of ferroptosis, we further evaluated ROS levels and the oxidative stress markers GSH and MDA. Our findings indicate that HIF-1α silencing and YZTLF treatment significantly decreased ROS and MDA levels in the IPF model while increasing GSH levels. Furthermore, the combined therapy exerted a synergistic antioxidant effect (**Figures 3L–O**). Furthermore, fibroblast proliferation, apoptosis, and fibrosis levels were examined. The results demonstrated that both HIF-1α silencing and YZTLF treatment exhibited pro-proliferative and anti-apoptotic effects, with the combined treatment showing enhanced efficacy (**Figures 3P–R**). Regarding fibrosis, both interventions displayed anti-fibrotic effects; however, YZTLF treatment demonstrated superior efficacy. This was evidenced by the expression levels of α-SMA and Collagen I in fibroblasts (**Figures 3S, T**).

### Mechanistic validation of HIF-1α/LSH/SCD1 regulatory dynamics in MRC-5 cell

**Figure 4:**
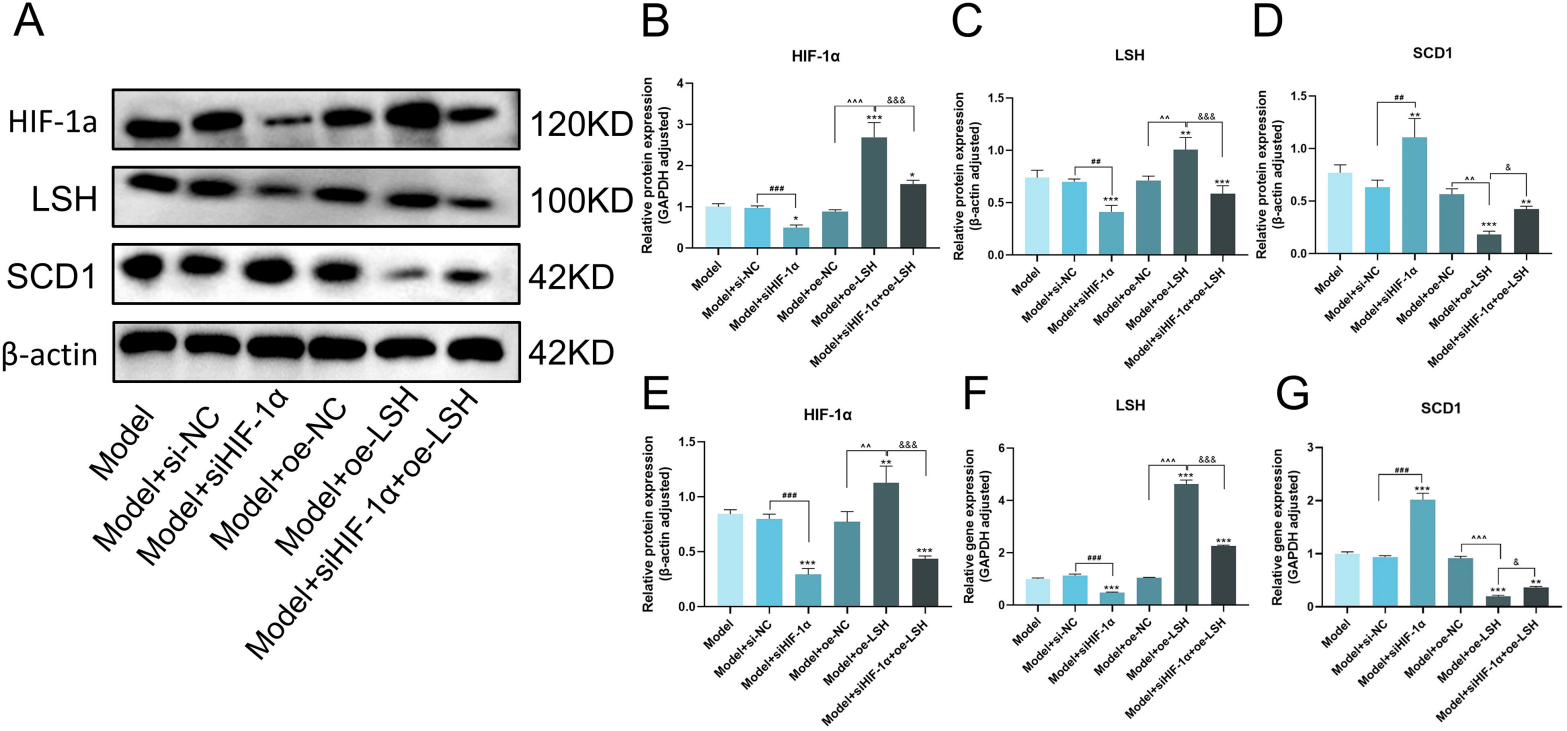
HIF-1α regulates SCD1 through LSH in IPF cell model. **(A)** Gray scale images of HIF-1α, LSH and SCD1 proteins. **(B–D)** Quantitative results of HIF-1α, LSH and SCD1 proteins. **(E–G)** mRNA expression levels of HIF-1α, LSH and SCD1. Statistical significance is indicated as follows: *p < 0.05, **p < 0.01, ***p < 0.001 vs. Model group; ^#^*p* < 0.05, ^##^*p* < 0.01, ^###^*p* < 0.001, Model+siNC vs. Model+siHIF-1α group; ^*p* < 0.05, ^^*p* < 0.01, ^^^*p* < 0.001, Model+oe-NC vs. Model+oe-LSH group; ^&^*p* < 0.05, ^&&^*p* < 0.01, ^&&&^*p* < 0.001, Model+oe-LSH vs. Model+siHIF-1α+oe-LSH group. Data are presented as mean ± SD (*n = 3*).

To further elucidate the interplay among LSH, HIF-1α, and SCD1 in IPF pathogenesis, we conducted functional studies using HIF-1α knockdown and LSH overexpression. Western blot and qPCR analyzes revealed markedly elevated HIF-1α levels in the model, model+si-NC, and model+oe-NC groups. HIF-1α knockdown significantly reduced its expression, while LSH overexpression also increased HIF-1α levels, likely due to ROS accumulation from exacerbated ferroptosis activating HIF-1α stabilization (24, 25). Combined HIF-1α silencing and LSH overexpression partially reversed this effect and the HIF-1α level was higher than alone HIF-1α silencing, confirming a regulatory feedback loop (**Figures 4A, B**).

LSH protein and mRNA levels were substantially upregulated in the model group, with further elevation observed in the model+oe-LSH group. HIF-1α knockdown significantly suppressed LSH expression, demonstrating HIF-1α-dependent positive regulation of LSH (**Figures 4C, F**). Conversely, SCD1 levels were reduced in the model group but increased upon HIF-1α silencing. LSH overexpression suppressed SCD1 expression, while dual intervention (siHIF-1α + oe-LSH) restored SCD1 levels, confirming LSH-mediated negative regulation of SCD1 and HIF-1α regulates SCD1 through LSH (**Figures 4D, G**).

### HIF-1α/LSH/SCD1 axis regulates ferroptosis and apoptosis in IPF cell model

**Figure 5:**
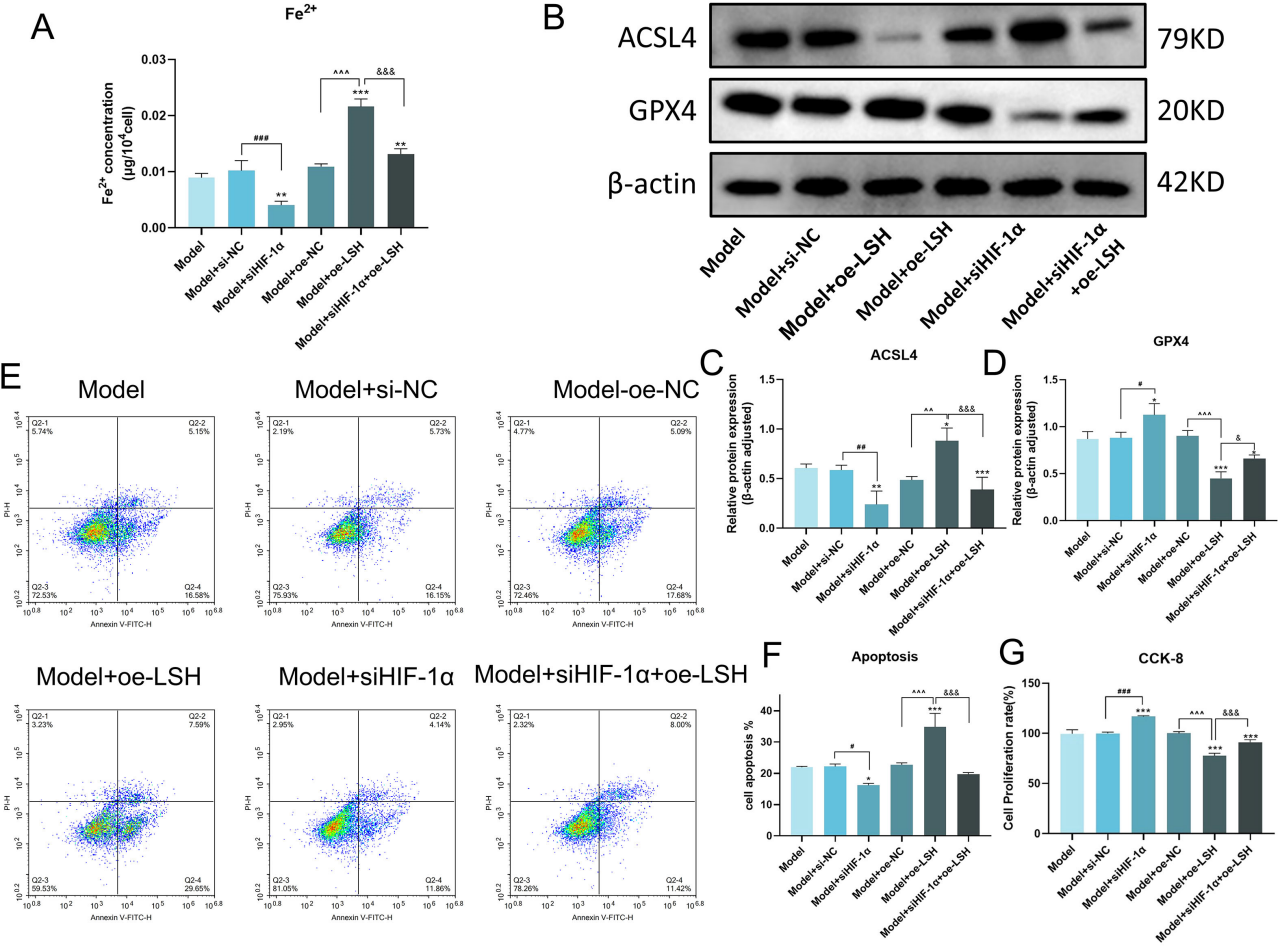
The HIF-1α/LSH/SCD1 axis mediates ferroptosis, apoptosis, and cell viability in the IPF cell model. **(A)** The level of Iron ion. **(B)** Representative western blot images of ACSL4 and GPX4 proteins. **(C, D)** Quantitative results of ACSL4 and GPX4 protein expression. **(E)** Distribution of apoptosis in MRC-5 cells. The quadrants indicate necrotic cells (Q2-1), early, apoptotic cells (Q2-2), viable cells (Q2-3), and late apoptotic cells (Q2-4). **(F)** Histogram quantifying the percentage of apoptotic cells in each group. **(G)** Cell viability was detected by CCK8 assay. Statistical significance is indicated as follows: *p < 0.05, **p < 0.01, ***p < 0.001 vs. Model group; ^#^*p* < 0.05, ^##^*p* < 0.01, ^###^*p* < 0.001, Model+siNC vs. Model+siHIF-1α group; ^*p* < 0.05, ^^*p* < 0.01, ^^^*p* < 0.001, Model+oe-NC vs. Model+oe-LSH group; ^&^*p* < 0.05, ^&&^*p* < 0.01, ^&&&^*p* < 0.001, Model+oe-LSH vs. Model+siHIF-1α+oe-LSH group. Data are presented as mean ± SD (*n = 3*).

Iron ion levels, a direct indicator of ferroptosis, were elevated in the model group. HIF-1α knockdown reduced iron accumulation, whereas LSH overexpression exacerbated it, even counteracting the iron-lowering effect of siHIF-1α (**Figure 5A**). Consistently, ACSL4 expression mirrored iron ion trends, while GPX4 showed an inverse pattern, validating HIF-1α/LSH/SCD1 axis-mediated ferroptosis regulation (**Figures 5B-D**). Apoptosis assays revealed that HIF-1α silencing attenuated TGF-β-induced apoptosis in MRC-5 cells, whereas LSH overexpression amplified apoptotic rates. Notably, LSH overexpression reversed the anti-apoptotic effect of HIF-1α knockdown (**Figures 5E, F**). Cell viability assays corroborated these findings, with HIF-1α inhibition enhancing survival and LSH overexpression diminishing cellular activity (**Figure 5G**).

### Regulation of oxidative stress via the HIF-1α/LSH/SCD1 pathway

**Figure 6:**
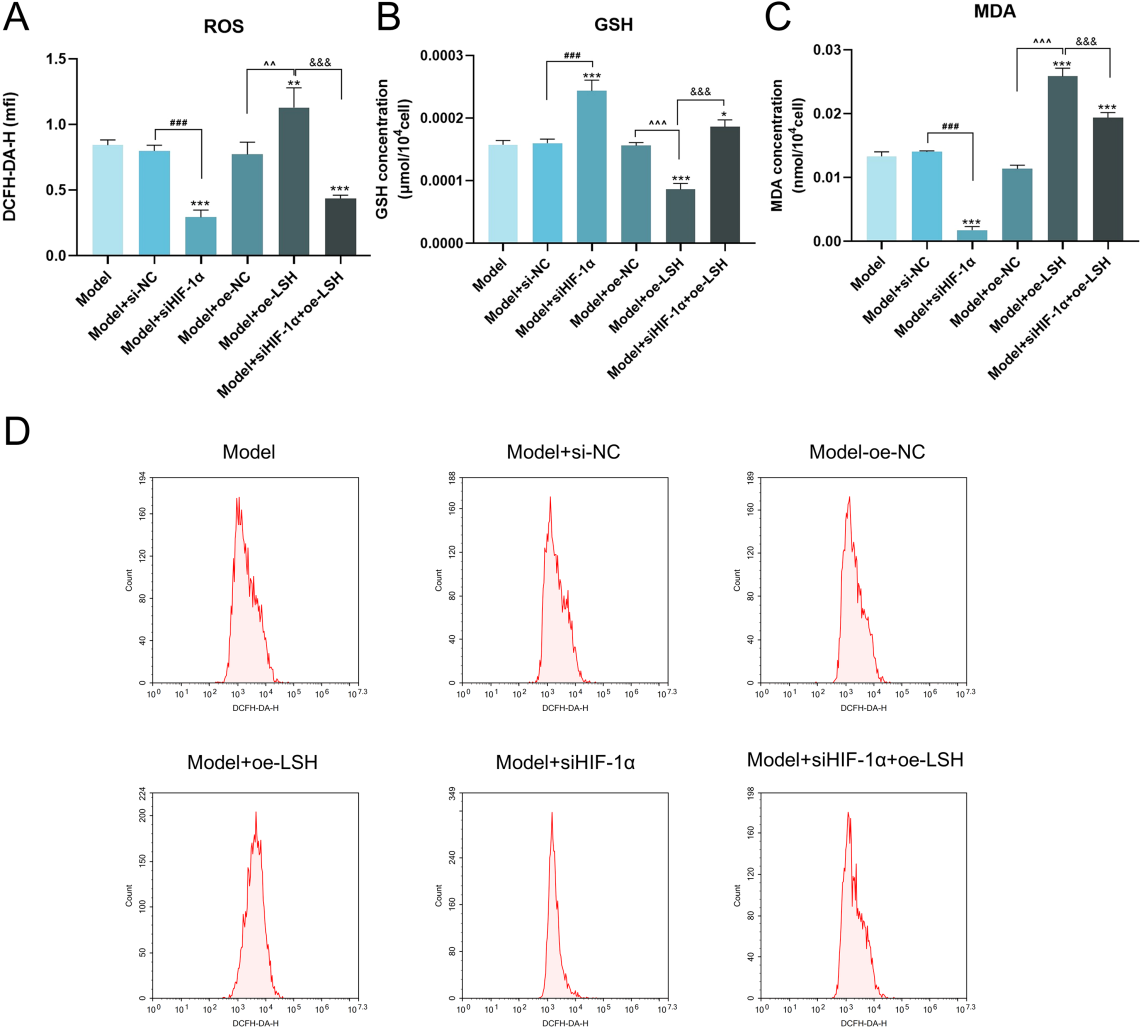
Oxidative stress is regulated through the HIF-1α/LSH/SCD1 pathway. **(A, B)** The level of ROS was detected by flow cytometry. **(C)** The level of GSH. **(D)** Representative flow cytometry histograms of intracellular ROS levels detected by DCFH-DA staining. Statistical significance is indicated as follows: *p < 0.05, **p < 0.01, ***p < 0.001 vs. Model group; ^#^*p* < 0.05, ^##^*p* < 0.01, ^###^*p* < 0.001, Model+siNC vs. Model+siHIF-1α group; ^*p* < 0.05, ^^*p* < 0.01, ^^^*p* < 0.001, Model+oe-NC vs. Model+oe-LSH group; ^&^*p* < 0.05, ^&&^*p* < 0.01, ^&&&^*p* < 0.001, Model+oe-LSH vs. Model+siHIF-1α+oe-LSH group. Data are presented as mean ± SD (*n = 3*).

Ferroptosis induces ROS-mediated lipid peroxidation, impairing autophagosome formation and recruitment of downstream proteins. HIF-1α silencing markedly decreased ROS levels, suggesting suppression of the HIF-1α/LSH/SCD1 pathway mitigates ROS accumulation during ferroptosis. Compared to the NC group, LSH overexpression elevated ROS levels, while HIF-1α knockdown attenuated this increase (**Figures 6A, D**). Oxidative stress markers also mirrored these trends, with MDA levels paralleling ROS elevation and GSH depletion, confirming their consistent regulation by the HIF-1α/LSH/SCD1 pathway (**Figures 6B, C**).

### Therapeutic efficacy of Yizong Tongluo formula *in vivo*

**Figure 7:**
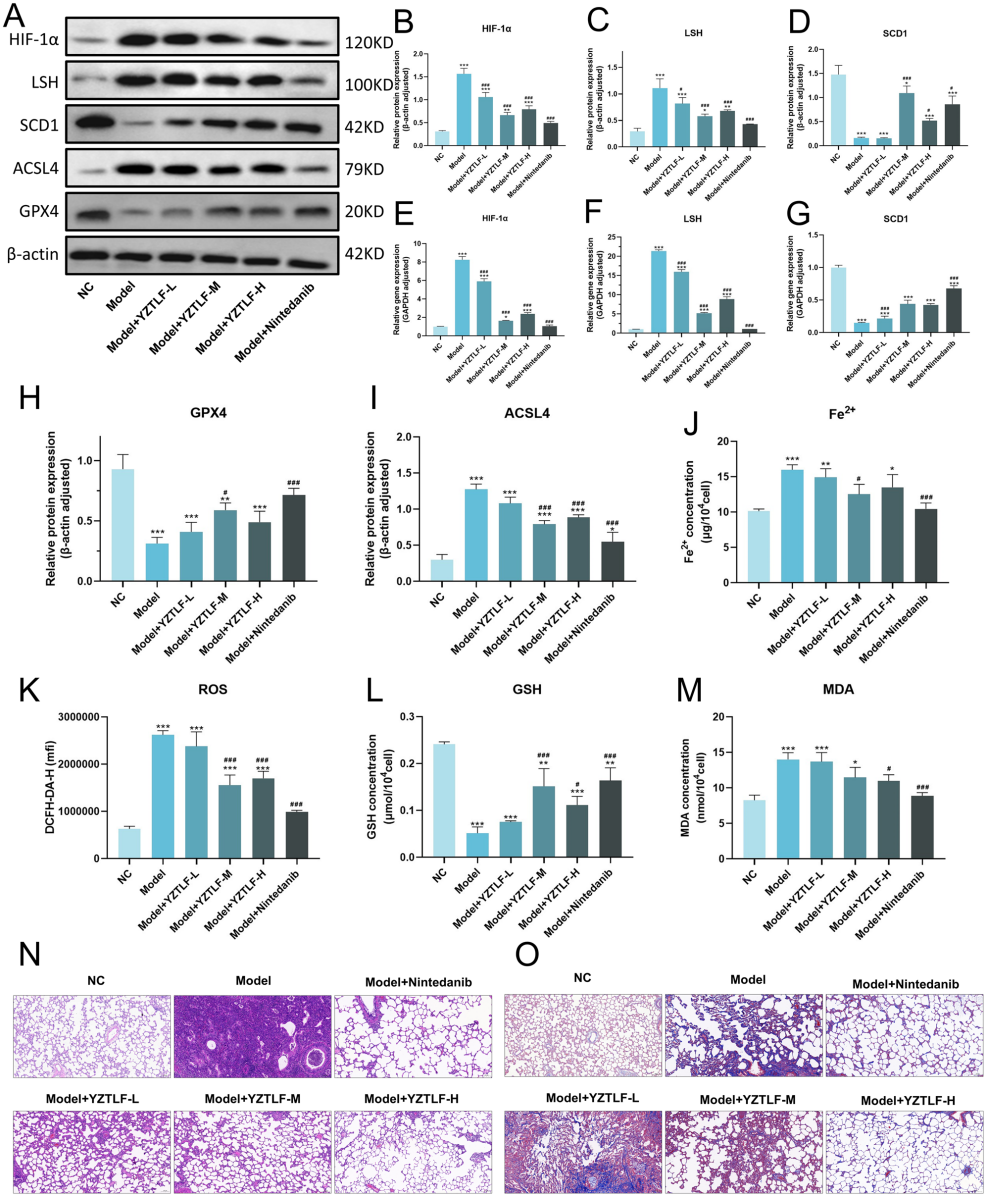
Yizong Tongluo Formula treated IPF through HIF-1α/LSH/SCD1 pathway in rat model. Scale bar = 100 μm. **(A)** Grayscale images of HIF-1α, LSH, SCD1, ACSL4 and GPX4 proteins. **(B–D)** Quantitative results of HIF-1α, LSH and SCD1 proteins. **(E–G)** mRNA expression levels of HIF-1α, LSH and SCD1. **(H, I)** Quantitative results of ACSL4 and GPX4 proteins. **(J)** The level of Iron ion. **(K)** The level of ROS. **(L, M)** The levels of GSH and MDA. **(N)** HE staining (scale bar, 100 μm). **(O)** Masson staining (scale bar, 100 μm). Statistical significance is indicated as follows: *p < 0.05, **p < 0.01, ***p < 0.001 vs. NC group; ^#^*p* < 0.05, ^##^*p* < 0.01, ^###^*p* < 0.001 vs. Model group. Data are presented as mean ± SD (*n = 6* for general parameters; *n = 3* for Western blot).

An intratracheal bleomycin-induced IPF rat model was established to evaluate YZTLF and nintedanib (50 mg/kg). Through 28 days, the body weight of rats was monitored as a general indicator of systemic health. The results showed that bleomycin induction caused significant weight loss compared to the control group. However, treatment with YZTLF and nintedanib effectively mitigated this weight reduction in a dose-dependent manner, suggesting a protective effect against systemic toxicity (**Supplementary Figure S1**). Nintedanib, a first-line tyrosine kinase inhibitor for IPF associated with diarrhea (discontinued in 5% of patients), served as the positive control (26). Bleomycin significantly increased HIF-1α and LSH protein/mRNA levels while reducing SCD1. YZTLF and nintedanib reversed these alterations, with high and medium dose groups showing superior therapeutic effects compared to the low-dose group. However, the high-dose group did not show a significantly greater effect than the medium-dose group, likely due to optimal concentration saturation (**Figures 7A–G**).

Bleomycin increased iron ion levels and ACSL4 expression while suppressing GPX4. Treatment with YZTLF and nintedanib attenuated these changes, reducing ferroptosis. Medium and high dose groups exhibited comparable efficacy, both outperforming the low-dose group, though nintedanib demonstrated stronger effects (**Figures 7H–J**). The levels of ROS and MDA in the lung tissue of rats treated with bleomycin increased significantly, while the level of GSH decreased significantly. The formula also alleviated pulmonary oxidative stress, normalizing elevated ROS and MDA levels and restoring GSH in bleomycin-treated rats (**Figures 7K–M**).

HE staining revealed collapsed alveolar structures, thickened septa, and inflammatory infiltration in the bleomycin group, indicative of lipid peroxidation and ferroptosis-driven membrane damage. Yizong Tongluo Formula improved pathology dose-dependently: low-dose partially reduced inflammation, medium-dose preserved alveolar integrity, and high-dose nearly reversed the damage caused by bleomycin. Nintedanib achieved optimal alveolar repair with minimal inflammation (**Figure 7N**). Masson staining showed extensive collagen deposition in the model group, reflecting fibroblast activation and ECM accumulation. Yizong Tongluo Formula dose-dependently reduced fibrosis, likely via SCD1-mediated membrane stabilization (**Figure 7O**).

## Discussion

Yizong Tongluo Formula demonstrates efficacy in treating IPF with Qi deficiency and blood stasis, however, its mechanism of action is less well-defined than those of established TCM formulations like Fufang Biejia Ruangan Pills (27), Shengxian Decoction (28), and Baihe Gujin Wan (29). To investigate its role in ferroptosis-related pulmonary fibrosis, we analyzed HIF-1α, LSH, SCD1, Fe^2+^, and ACSL4 in clinical samples. The formula shares components with other IPF-targeting TCMs, including *Codonopsis pilosula* from Fufang Biejia Ruangan Pills, *Astragalus membranaceus* from Shengxian Decoction, and *Platycodon grandiflorum* from Baihe Gujin Wan.

Steroids used to be considered a standard treatment for IPF, and often combined with immunosuppressants (30). But now, this therapy has been overturned, which demonstrated that triple therapy (prednisone, azathioprine, and N-acetylcysteine) was associated with increased risks of mortality and hospitalization compared to placebo (31). And current international guidelines strongly recommend against the routine use of corticosteroids for stable IPF, restricting their application primarily to the management of acute exacerbations (32). In recent clinical reports, with current therapeutic strategies primarily relying on the antifibrotic agents nintedanib and pirfenidone. Pivotal randomized controlled trials have demonstrated that these drugs can significantly reduce the rate of decline in FVC by approximately 50% over one year (26). However, nintedanib is frequently associated with gastrointestinal toxicity, particularly diarrhea and hepatic enzyme elevation (33), while pirfenidone often causes nausea, rash, and photosensitivity, leading to treatment discontinuation in a substantial proportion of patients (34). Compared with previous treatment modalities, YZTLF not only lacks these adverse effects, but also can potentially offer a safer therapeutic option or a complementary strategy to current standard-of-care regimens.

*Codonopsis pilosula* tonifies Qi, enhances immunity, and promotes hematopoietic regeneration (35, 36). *Astragalus membranaceus* activates blood circulation to counteract stasis, mitigates ischemic injury, and protects endothelial cells via antioxidative and anti-inflammatory pathways (37, 38). *Platycodon grandiflorum* resolves phlegm, suppresses cough, and reduces inflammation by inhibiting IGF-IIR to attenuate angiotensin II-induced apoptosis, thereby improving cardiovascular function (39). The formula aligns with TCM principles: *Astragalus* (monarch herb) replenishes thoracic Qi; *Codonopsis* and *Rhodiola* (minister herbs) tonify heart-spleen Qi and resolve blood stasis; *Cimicifuga* and *Platycodon* (assistant herbs) direct drug action to the lungs.

While TCM emphasizes holistic regulation, we investigated the molecular mechanisms of Yizong Tongluo Formula. Clinical and cellular studies confirmed its modulation of the HIF-1α/LSH/SCD1 pathway in IPF-associated ferroptosis and oxidative stress (40). In IPF, hypoxia stabilizes HIF-1α, which triggers alveolar epithelial apoptosis and disease progression. Dual-luciferase reporter assays and functional studies verified that HIF-1α binds to specific DNA regions of LSH, promoting LSH expression. LSH further suppresses SCD1, exacerbating ferroptosis and oxidative stress (41, 42). These findings align with our experimental data, though the formula’s multi-target nature suggests additional pathways require exploration.

Consistent with recent studies demonstrating that LSH activity is critically modulated by posttranslational modifications (43), we propose that LSH likely promotes SCD1 transcription by enhancing the chromatin accessibility at the SCD1 promoter region or by functioning as a transcriptional co-activator, thereby facilitating lipid desaturation and ferroptosis resistance.

Ferroptosis is increasingly recognized as a hallmark pathogenic feature shared across a broad spectrum of diseases characterized by iron dysregulation and oxidative stress. At the biochemical level, this lethal cascade is fundamentally driven by the accumulation of intracellular labile iron, which catalyzes the Fenton and Haber-Weiss reactions (44). Iron-dependent cytotoxicity is extensively documented in hepatic pathologies. In hereditary hemochromatosis, genetic defects lead to systemic iron overload, triggering hepatocyte ferroptosis and subsequent tissue fibrosis (45). Similarly, recent studies have implicated ferroptosis in the pathogenesis of drug-induced liver injury (DILI) (46), particularly in acetaminophen overdose and in liver injury induced by heavy metal exposure, where environmental toxins disrupt iron homeostasis and antioxidant defenses (47). Ferroptosis and oxidative stress are also central pathological mechanisms in IPF. Recurrent alveolar epithelial injury and aberrant repair initiate IPF progression, with ferroptosis representing a critical mode of epithelial cell death (48, 49). Targeting ferroptosis inhibition thus emerges as a viable therapeutic strategy. In IPF with Qi deficiency and blood stasis, systemic hypoxia exacerbates ROS/HIF-1α-associated oxidative stress and inflammatory responses, driving fibrotic remodeling (50). Concurrently, iron overload catalyzes ROS generation via the Fenton reaction, directly inducing lipid peroxidation. Yizong Tongluo Formula mitigates these processes by modulating the HIF-1α/LSH/SCD1 pathway, offering a mechanism-based intervention for this IPF with Qi deficiency and blood stasis.

Although it has been reported that certain TCM components such as Bupleurum chinense exhibited potential hepatotoxicity in high doses or specific extraction contexts (51), the YZTLF is formulated based on the TCM principle of ‘Jun-Chen-Zuo-Shi’ (Monarch-Minister-Assistant-Guide), which emphasizes balanced synergy and detoxification. Notably, the major components of YZTLF, including *Astragalus membranaceus, Codonopsis pilosula, Rhodiola rosea* and *Angelica sinensis*, have been extensively documented to possess significant hepatoprotective properties (52–54). In our study, we strictly controlled the quality and dosage of herbs. Furthermore, no adverse hepatic events were observed in our clinical cohort, and the rats in the treatment groups showed no signs of systemic toxicity, such as significant weight loss or behavioral abnormalities.

Compared with previous studies, this work uniquely identifies the HIF-1α/LSH/SCD1 axis as a molecular bridge connecting the TCM syndrome of ‘Qi deficiency and blood stasis’ with ferroptosis-driven lipid peroxidation. Unlike conventional anti-fibrotic research, our findings provide a precise biological explanation for how YZTLF remodels the hypoxic microenvironment caused by blood stasis. However, there are some limitations in this study. First, we evaluated the efficacy of the whole formula without identifying specific bioactive monomers via metabolomics, which remains a critical direction for future research to clarify the material basis. Second, the clinical sample size was relatively small, and the bleomycin-induced acute model may not fully represent the chronic progressive nature of human IPF. Furthermore, pulmonary fibrosis is a complex process involving the interplay of multiple cell types. While this study primarily focused on fibroblast activation to elucidate the anti-fibrotic mechanism of YZTLF, we acknowledge that alveolar epithelial injury is a crucial initiating event in IPF pathogenesis. The absence of data regarding epithelial cells limits our comprehensive understanding of the formula’s potential protective role during the disease initiation phase.

Clinical samples from IPF patients with Qi deficiency and blood stasis showed elevated iron ions, ACSL4, and reduced GPX4, confirming ferroptosis activation. Aberrant HIF-1α, LSH, and SCD1 protein and mRNA levels were also observed. ChIP assays verified HIF-1α binding to specific DNA regions of LSH, while dual-luciferase reporter assays and functional studies confirmed LSH-mediated suppression of SCD1. Yizong Tongluo Formula suppressed ferroptosis via HIF-1α/LSH/SCD1 modulation, as validated by HIF-1α silencing and LSH overexpression experiments. Animal studies further confirmed the formula’s capacity to attenuate ferroptosis, oxidative stress, and pulmonary fibrosis via the HIF-1α/LSH/SCD1 pathway.

**Figure 8:**
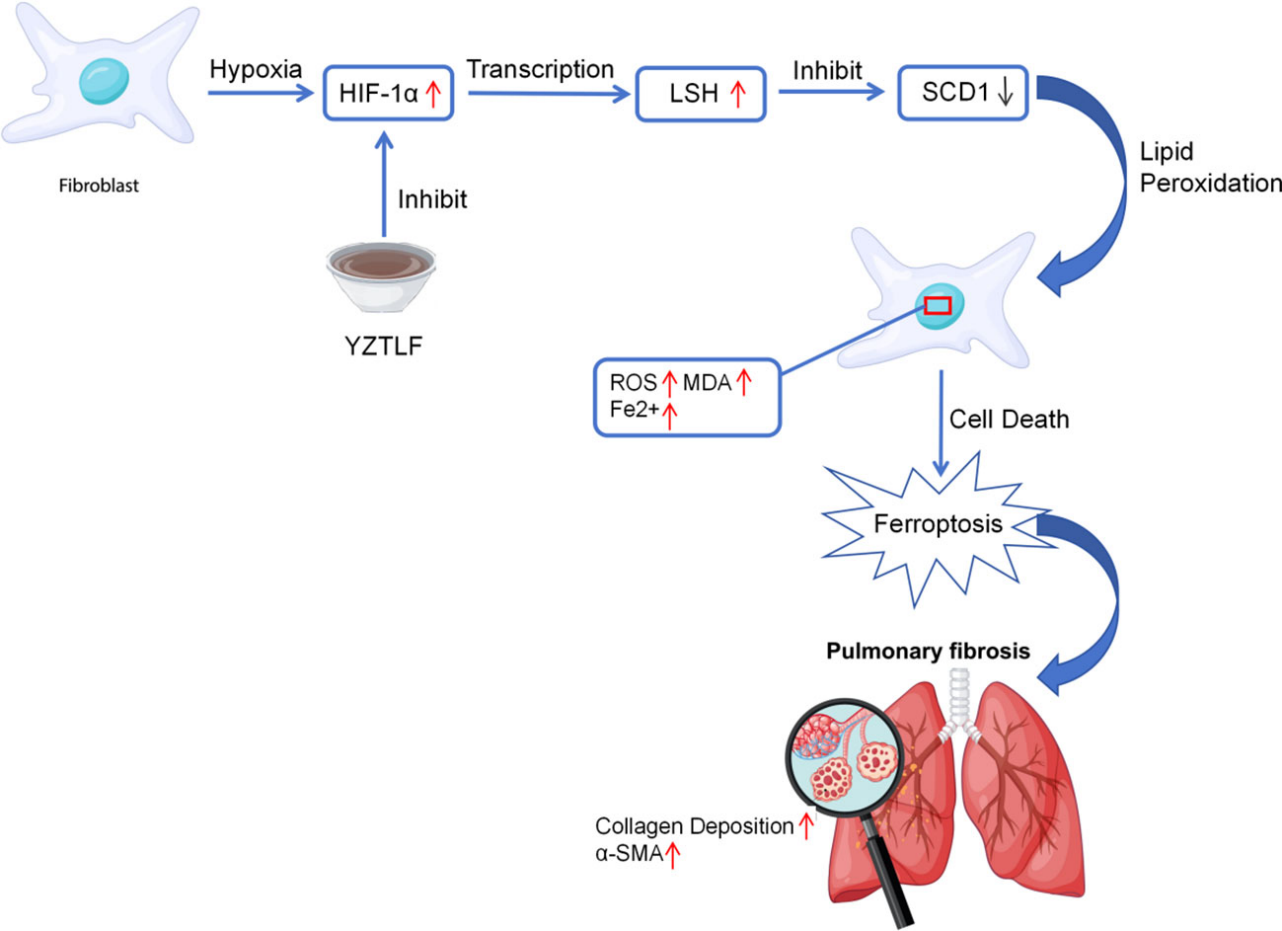
Schematic diagram illustrating the therapeutic mechanism of YZTLF in IPF. Under hypoxic conditions, HIF-1α accumulates and transcriptionally activates LSH. Upregulated LSH acts as a repressor of SCD1, leading to the downregulation of SCD1 expression. This loss of SCD1 triggers lipid peroxidation (accumulation of ROS, MDA, and Fe^2+^), driving the cells toward ferroptosis. The ferroptotic cell death of fibroblasts ultimately promotes collagen deposition and pulmonary fibrosis. YZTLF exerts its anti-fibrotic effect by inhibiting HIF-1α, thereby interrupting this signaling axis, restoring SCD1 levels, and preventing ferroptosis-mediated fibrosis.

In summary, our study provides that YZTLF exerts a potent anti-fibrotic effect by targeting the HIF-1α/LSH/SCD1 signaling axis. As illustrated in **Figure 8**, YZTLF inhibits hypoxia-induced HIF-1α accumulation, thereby suppressing LSH expression and restoring SCD1 levels. This restoration of SCD1 prevents lipid peroxidation and ferroptosis in fibroblasts, ultimately attenuating collagen deposition and pulmonary fibrosis.

## Data Availability

The original contributions presented in the study are included in the article/**Supplementary Material**. Further inquiries can be directed to the corresponding author.
