# Supplementary material for: Yizong Tongluo formula attenuates idiopathic pulmonary fibrosis and inflammatory injury by inhibiting HIF-1α/LSH/SCD1-mediated ferroptosis
Source: Front Immunol. 2026 Feb 11;17:1760615. doi: 10.3389/fimmu.2026.1760615 (PMC12932185; doi:10.3389/fimmu.2026.1760615)
Supplement: Supplementary file 1 [file DataSheet1.docx]

Body Weight Changes of Rat


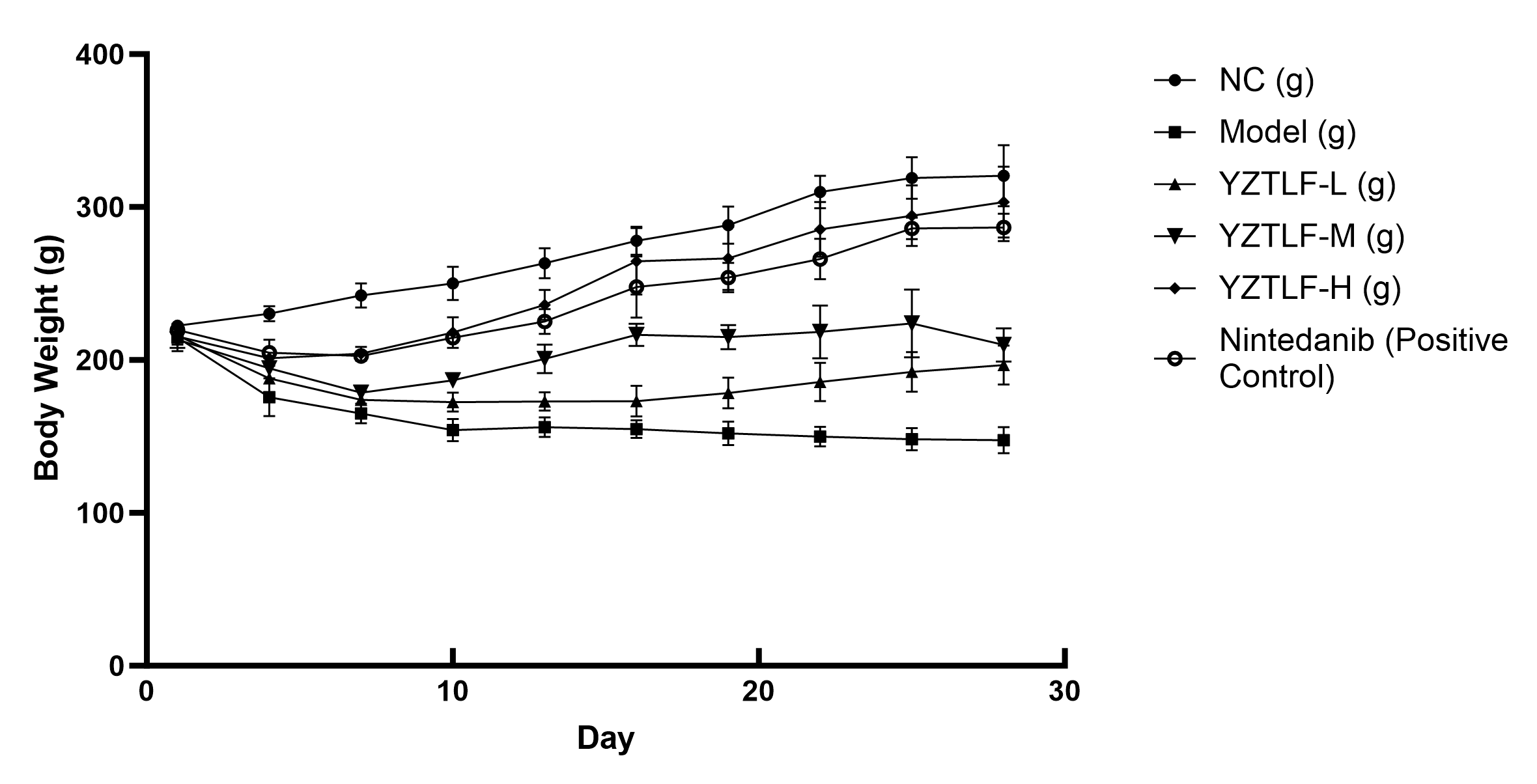


Figure S1 Body weight changes of rats in each group (g).

NC Group

| **Day** | **Rat 1** | **Rat 2** | **Rat 3** | **Rat 4** | **Rat 5** | **Rat 6** |
| --- | --- | --- | --- | --- | --- | --- |
| 1 | 220 | 224 | 224 | 225 | 220 | 222 |
| 4 | 221 | 235 | 232 | 234 | 230 | 230 |
| 7 | 240 | 246 | 235 | 249 | 232 | 252 |
| 10 | 242 | 260 | 256 | 256 | 255 | 232 |
| 13 | 265 | 275 | 249 | 270 | 267 | 254 |
| 16 | 281 | 289 | 263 | 283 | 280 | 272 |
| 19 | 280 | 304 | 280 | 297 | 273 | 295 |
| 22 | 290 | 321 | 315 | 312 | 313 | 308 |
| 25 | 295 | 325 | 330 | 328 | 326 | 310 |
| 28 | 299 | 345 | 323 | 343 | 301 | 312 |

Model Group (Bleomycin)

| **Day** | **Rat 1** | **Rat 2** | **Rat 3** | **Rat 4** | **Rat 5** | **Rat 6** |
| --- | --- | --- | --- | --- | --- | --- |
| 1 | 214 | 198 | 221 | 217 | 215 | 224 |
| 4 | 188 | 179 | 171 | 162 | 191 | 163 |
| 7 | 159 | 174 | 163 | 171 | 165 | 158 |
| 10 | 162 | 143 | 157 | 148 | 160 | 155 |
| 13 | 159 | 167 | 153 | 150 | 157 | 150 |
| 16 | 157 | 161 | 150 | 161 | 153 | 147 |
| 19 | 160 | 157 | 148 | 157 | 151 | 139 |
| 22 | 150 | 160 | 146 | 153 | 149 | 141 |
| 25 | 151 | 159 | 144 | 151 | 146 | 138 |
| 28 | 153 | 159 | 142 | 152 | 143 | 136 |

YZTLF-L (Low Dose)

| **Day** | **Rat 1** | **Rat 2** | **Rat 3** | **Rat 4** | **Rat 5** | **Rat 6** |
| --- | --- | --- | --- | --- | --- | --- |
| 1 | 217 | 208 | 222 | 224 | 213 | 213 |
| 4 | 196 | 185 | 200 | 202 | 170 | 175 |
| 7 | 168 | 168 | 180 | 182 | 173 | 173 |
| 10 | 170 | 164 | 176 | 182 | 170 | 173 |
| 13 | 180 | 165 | 176 | 178 | 168 | 170 |
| 16 | 176 | 159 | 169 | 190 | 173 | 171 |
| 19 | 177 | 161 | 189 | 188 | 179 | 176 |
| 22 | 177 | 169 | 197 | 203 | 185 | 183 |
| 25 | 180 | 176 | 205 | 209 | 193 | 190 |
| 28 | 181 | 185 | 199 | 217 | 200 | 198 |

YZTLF-M (Medium Dose)

| **Day** | **Rat 1** | **Rat 2** | **Rat 3** | **Rat 4** | **Rat 5** | **Rat 6** |
| --- | --- | --- | --- | --- | --- | --- |
| 1 | 219 | 207 | 210 | 220 | 210 | 214 |
| 4 | 197 | 190 | 191 | 202 | 192 | 196 |
| 7 | 181 | 175 | 175 | 185 | 176 | 180 |
| 10 | 189 | 182 | 184 | 193 | 183 | 189 |
| 13 | 200 | 193 | 197 | 219 | 194 | 201 |
| 16 | 213 | 224 | 226 | 216 | 207 | 213 |
| 19 | 207 | 217 | 224 | 224 | 207 | 211 |
| 22 | 210 | 230 | 238 | 233 | 203 | 197 |
| 25 | 211 | 243 | 252 | 234 | 205 | 198 |
| 28 | 200 | 211 | 208 | 230 | 201 | 209 |

YZTLF-H (High Dose)

| **Day** | **Rat 1** | **Rat 2** | **Rat 3** | **Rat 4** | **Rat 5** | **Rat 6** |
| --- | --- | --- | --- | --- | --- | --- |
| 1 | 212 | 215 | 215 | 213 | 218 | 220 |
| 4 | 198 | 200 | 201 | 198 | 204 | 206 |
| 7 | 203 | 198 | 207 | 200 | 208 | 209 |
| 10 | 220 | 199 | 225 | 215 | 225 | 224 |
| 13 | 218 | 240 | 243 | 231 | 243 | 241 |
| 16 | 224 | 278 | 282 | 279 | 266 | 258 |
| 19 | 225 | 276 | 279 | 267 | 277 | 275 |
| 22 | 250 | 294 | 297 | 284 | 294 | 293 |
| 25 | 257 | 295 | 293 | 300 | 311 | 310 |
| 28 | 274 | 289 | 286 | 316 | 328 | 327 |

Nintedanib (Positive Control)

| **Day** | **Rat 1** | **Rat 2** | **Rat 3** | **Rat 4** | **Rat 5** | **Rat 6** |
| --- | --- | --- | --- | --- | --- | --- |
| 1 | 219 | 220 | 219 | 217 | 221 | 222 |
| 4 | 205 | 190 | 210 | 215 | 201 | 208 |
| 7 | 201 | 202 | 201 | 202 | 200 | 210 |
| 10 | 212 | 218 | 213 | 213 | 215 | 217 |
| 13 | 215 | 233 | 228 | 228 | 232 | 215 |
| 16 | 213 | 248 | 265 | 243 | 270 | 247 |
| 19 | 244 | 240 | 258 | 258 | 262 | 262 |
| 22 | 272 | 242 | 273 | 273 | 259 | 277 |
| 25 | 287 | 273 | 288 | 285 | 292 | 292 |
| 28 | 288 | 270 | 285 | 295 | 293 | 289 |
